# Supplementary material for: An intelligent optimization method for highway route selection based on comprehensive weight and TOPSIS
Source: PLoS One. 2022 Feb 25;17(2):e0262588. doi: 10.1371/journal.pone.0262588 (PMC8880847; doi:10.1371/journal.pone.0262588)
Supplement: S1 Appendix — (DOCX) [file pone.0262588.s001.docx]

Appendix A: Theorem 1

**Theorem 1**: If $\sum_{i=1}^{m} z_{ij}^{2}>0$（*j=1,2, …,n*）, then the optimization formula (15) has a unique solution, which is

| $\omega=\left[ \rho\eta_{1}+\left( 1-\rho\right)\mu_{1},\rho\eta_{2}+\left( 1-\rho\right)\mu_{2},\cdots,\rho\eta_{n}+\left( 1-\rho\right)\mu_{n} \right]^{T}$ | (16) |
| --- | --- |

**Proof**  We have the Lagrange function:

| $L\left( W,\lambda\right)=\sum_{i=1}^{m} \sum_{j=1}^{n} \left\{ \rho\left[ \left( \omega_{j}-\mu_{j} \right)z_{ij} \right]^{2}+\left( 1-\rho\right)\left[ \left( \omega_{j}-\eta_{j} \right)z_{ij} \right]^{2} \right\}+2\lambda\left( \sum_{j=1}^{n} \omega_{j}-1 \right)$ | (26) |
| --- | --- |

According to the first-order condition (a necessary condition) for the existence of an extreme value, let

| $\left\{ \begin{aligned} &\frac{\partial L}{\partial\omega_{j}}=\sum_{i=1}^{m} 2\rho\left[ \left( \omega_{j}-\mu_{j} \right)z_{ij}^{2} \right]+2\left( 12\rho\right)\left[ \left( \omega_{j}-\eta_{j} \right)z_{ij}^{2} \right]+2\lambda=0 \\ &\frac{\partial L}{\partial\lambda}=2\left\vert\sum_{j=1}^{n} \omega_{j}-1 \right\vert=0,j=1,2,\cdots,n \end{aligned} \right.$ | (27) |
| --- | --- |

Simplifying, we obtain

| $\left\vert\begin{matrix} B_{nn} & e_{n1} \\ e_{m1}^{T} & 0 \end{matrix} \right\vert\left\vert\begin{matrix} \omega_{n1} \\ \lambda\end{matrix} \right\vert=\left\vert\begin{matrix} C_{n1} \\ 1 \end{matrix} \right\vert$ | (28) |
| --- | --- |

This is a system of equations composed of n+1 variables and n+1 equations. It is expressed by the matrix

| $\left\vert\begin{matrix} B_{nn} & e_{n1} \\ e_{n1}^{T} & 0 \end{matrix} \right\vert\cdot\left\vert\begin{aligned} &\omega_{n1} \\ &\lambda\end{aligned} \right\vert=\left\vert\begin{aligned} &C_{n1} \\ &1 \end{aligned} \right\vert$ | (29) |
| --- | --- |

where

$$B_{nn}=diag\left| \sum_{i=1}^{m} z_{i1}^{2},\sum_{i=1}^{m} z_{i2}^{2},\cdots,\sum_{i=1}^{m} z_{in}^{2} \right|$$

$$e_{n1}=\left( 1,1,\cdots,1 \right)^{T}$$

$$\omega_{n1}=\left( \omega_{1},\omega_{2},\cdots,\omega_{n} \right)^{T}$$

$$C_{n1}=\left| \sum_{i=1}^{m} \left[ \rho\eta_{1}+\left( 1-\rho\right)\mu_{1} \right]z_{i1}^{2},\sum_{i=1}^{m} \left[ \rho\eta_{2}+\left( 1-\rho\right)\mu_{2} \right]z_{i2}^{2},\cdots,\sum_{i=1}^{m} \left[ \rho\eta_{n}+\left( 1-\rho\right)\mu_{n} \right]z_{in}^{2} \right|$$

According to the second-order conditions (sufficient conditions) for the existence of an extremum,

| $\frac{\boldsymbol{\partial}^{\boldsymbol{2}}\boldsymbol{L}}{\boldsymbol{\partial}\boldsymbol{\omega}_{\boldsymbol{j}}^{\boldsymbol{2}}}\boldsymbol{=}\sum_{\boldsymbol{j=1}}^{\boldsymbol{m}} \boldsymbol{2}\boldsymbol{z}_{\boldsymbol{ij}}^{\boldsymbol{2}}$,  $\frac{\boldsymbol{\partial}^{\boldsymbol{2}}\boldsymbol{L}}{\boldsymbol{\partial}\boldsymbol{\omega}_{\boldsymbol{j}}\boldsymbol{\partial\lambda}}\boldsymbol{=2}$,  $\frac{\boldsymbol{\partial}^{\boldsymbol{2}}\boldsymbol{L}}{\boldsymbol{\partial}\boldsymbol{\omega}_{\boldsymbol{j}}\boldsymbol{\partial}\boldsymbol{\omega}_{\boldsymbol{j}}}\boldsymbol{=0}$,  $\frac{\boldsymbol{\partial}^{\boldsymbol{2}}\boldsymbol{L}}{\boldsymbol{\partial}\boldsymbol{\lambda}^{\boldsymbol{2}}}\boldsymbol{=0}$ | (30) |
| --- | --- |

Let

| $D_{k}=\left\vert\begin{matrix} \sum_{i=1}^{m} z_{i1}^{2} & 0 & \cdots& 0 \\ 0 & \sum_{i=1}^{m} z_{i2}^{2} & \cdots& 0 \\ \vdots& \vdots& & \vdots\\ 0 & 0 & \cdots& \sum_{i=1}^{m} z_{ik}^{2} \end{matrix} \right\vert$, k=1,2,…,n | (31) |
| --- | --- |

Therefore, when D_k_＞0 (k=1,2,…,m), that is, when $\sum_{i=1}^{m} z_{ij}^{2}>0$ (*j=1, 2, … ,m*), the model described in (15) must have solutions. Equation (15) is solved as follows:

From formula (28),

| $\left\vert\begin{aligned} &B_{nn}W_{n1}+\lambda e_{n1}=C_{n1} \\ &e_{m1}^{T}W_{n1}=1 \end{aligned} \right.$ | (32) |
| --- | --- |

Because *B_mm_* is invertible, i.e., *B^-1^_mm_* exists, the solution of the matrix equation (13) yields

| $W_{n1}=B_{nn}^{-1}\left\vert C_{n1}+\frac{1-e_{n1}^{T}B_{nn}^{-1}C_{n1}}{e_{n1}^{T}B_{nn}^{-1}e_{n1}}e_{n1} \right\vert$ | (33) |
| --- | --- |

Again, because

| $B_{nn}^{-1}C_{n1}=\left[ \rho\eta_{1}+\left( 1-\rho\right)\mu_{1},\rho\eta_{2}+\left( 1-\rho\right)\mu_{2},\cdots,\rho\eta_{n}+\left( 1-\rho\right)\mu_{n} \right]^{T}$  $e_{n1}^{T}B_{nn}^{-1}C_{n1}=\sum_{j=1}^{n} \left[ \rho\eta_{j}+(11\rho)\mu_{j} \right]=1$ | (34) |
| --- | --- |

we have,

| $W_{n1}=B_{nn}^{-1}C_{n1}=\left[ \rho\eta_{1}+\left( 1-\rho\right)\mu_{1},\rho\eta_{2}+\left( 1-\rho\right)\mu_{2},\cdots,\rho\eta_{n}+\left( 1-\rho\right)\mu_{n} \right]^{T}$ | (35) |
| --- | --- |

Thus, the theorem is proven.
