# Supplementary material for: An intelligent optimization method for highway route selection based on comprehensive weight and TOPSIS
Source: PLoS One. 2022 Feb 25;17(2):e0262588. doi: 10.1371/journal.pone.0262588 (PMC8880847; doi:10.1371/journal.pone.0262588)
Supplement: Attachment — Submitted filename: Response to Reviewers.doc [file pone.0262588.s003.doc]

# Response to Reviewer’s Comments

# Reviewer #1:

Point 1: The results of the research should be in the abstract. Materials and Methods should be corrected for example see the following papers:

We revised the reference documents and improved the materials and methods.

Pamucar, D., & Ćirović, G. (2018). Vehicle route selection with an adaptive neuro fuzzy inference system in uncertainty conditions. Decision Making: Applications in Management and Engineering, 1 (1), 13-37.

Liu, F., Aiwu, G., Lukovac, V., & Vukic, M. (2018). A multicriteria model for the selection of the transport service provider: A single valued neutrosophic DEMATEL multicriteria model. Decision Making: Applications in Management and Engineering, 1 (2), 121-130.

Noureddine, M., & Ristic, M. (2019). Route planning for hazardous materials transportation: Multicriteria decision making approach. Decision Making: Applications in Management and Engineering, 2 (1), 66-85.

Blagojević, A., Vesković, S., Kasalica, S., Gojić, A., & Allamani, A. (2020). The application of the fuzzy AHP and DEA for measuring the efficiency of freight transport railway undertakings. Operational Research in Engineering Sciences: Theory and Applications, 3 (2), 1-23.

Kasalica, S., Obradović, M., Blagojević, A., Jeremić, D., & Vuković, M. (2020). Models for ranking railway crossings for safety improvement. Operational Research in Engineering Sciences: Theory and Applications, 3 (3), 84-100.

Response：We revised the References and improved the Materials and Methods.

Point 2: Algorithms cannot be results. Algorithms are a paper methodology. Correct this and better explain the methodology. The selection Discussion and empirical research is debatable. First is the Case study, then the results. There is no discussion. The results are incomplete. One result must be obtained with the TOPSIS method, and then a sensitivity analysis must be performed, in order to exclude some criteria, etc. It is necessary to make scenarios and then discuss all these results.

Response：We have updated the Discussion and empirical research. The sensitivity analysis with variable Rho is added base on the comments. After analysis, the variable Rho directly affects the closeness and project.

Point 3: The conclusion should include the most important results, research limits and guidelines for future research.

Response：We rewrote the conclusions and added research results, research limitations and guidelines for future research.

Point 4: Adjust the paper according to the guidelines.

Response：Revised according to comments.

# Reviewer #2:

Point 1: I suggest the authors to improve the introduction section. Authors should better highlight the objective of their work and to what extent it contributes to close a gap in the existing literature and/or practice. What is the innovative value of the contribution proposed by the authors?

Response：We rewrote the Introductions, highlight the objectives of previous work and the extent to which it contributes to bridging gaps in existing literature and practice.

Point 2: In introduction section authors should provide more information about existing MCDM models used in the field and their benefits/weaknesses.

Response：Revised according to comments.

Point 3: Why you have used TOPSIS method? Why not RAFSI, MABAC, MAIRCA, VIKOR methdos? This should be discussed. The authors need to discuss their contributions compared to those in related papers. The authors must clearly discuss the significance of the research problem in the first section.

Response：Revised according to comments. It is supplemented in Introduction.

Point 4: Add separate literature review section. You should provide more recent references published in last two-three years. Remove references published before 2017.

Response：Revised according to comments. Several references have been added and removed some references published before 2017.

Point 5: The authors should explain why they insist in practically using the Comprehensive weight model. Why not LBWA, FUCOM or BWM models?

Response：Revised according to comments. It is supplemented in TOPSIS Evaluation Model. LBWA, FUCOM or BWM models can also be used. However, considering that TOPSIS is simpler and can make full use of the existing data, it is more suitable for internal sorting of sample data, this paper adopts TOPSIS.

Point 6: You should provide step by step calculations for provided methodology, especially Comprehensive weight model. You should explain in detail this methodology.

Response：In this paper, Materials and Methods are the whole process of calculation, which is proved in Appendix A. We also added the explanation of preference coefficient according to the Comments.

Point 7: Explain in more details in the data used in the case study, the data for the testing, the criterion for the accuracy, and others to claim these points.

Response：Revised according to comments. It is supplemented in Discussion and empirical research.

Point 8: Validation section is missing. How we can judge about these results? Comparisons with existing algorithms from the literature is missing.

Response：Revised according to comments. The comprehensive weight vector increases the preference coefficient ρ, which is essential different from existing other comprehensive weight methods. T

Point 9: Discussion section is missing. How should we know about the quality of these solutions? Could you compare these results with some existing approaches in literature? The improvement must be discussed.

Response：Revised according to comments. The comprehensive weight vector increases the preference coefficient ρ, which is essential different from existing other comprehensive weight methods. The preference coefficient ρ which can be determined by the accuracy of subjective and objective information and preference. When the decision-maker obtains the subjective weight and objective weight, through comprehensive analysis of the project, a preference coefficient from the perspective of the interests of the decision-maker is proposed, which can better serve the decision-maker. Intelligent computing can be used to form an evaluation of the scheme by a large number of evaluation groups (including experts, stakeholders, etc.), and the evaluation results are more objective.

Point 10: The conclusion section seems to rush to the end. The authors will have to demonstrate the impact and insights of the research. The authors need to clearly provide several solid future research directions. Clearly state your unique research contributions in the conclusion section. Add limitations of the model. No bullets should be used in your conclusion section.

Response：Revised according to comments.

# Reviewer #3:

Point: 1.It is suggested that the authors introduce the organization of this paper in the end of introduction.

Response：Revised according to comments.

Point: 2. Page 9, line 5, “and each scheme m has a quantitative value B_{ij}” should be “and each scheme has a quantitative value b_{ij} for each quantitative index”.

Response: We have modified for “and each scheme has a quantitative value Bij for each quantitative index” ( Line 24 ).

Point: 3. Page 10, line 16, “and f_i^k is the importance of indicator F_i^k compared with indicator F_j^k” should be “and f_{ij}^k is the importance of indicator F_i^k compared with indicator F_j^k”.

Response: We have modified for “and f_{ij}^k is the importance of indicator F_i^k compared with indicator F_j^k”.” ( Line 24 ).

Point: 4. Page 11, formula (7), what does the symbols “x_{ij}, max(x_j), min(x_j)” stand for? Maybe they are “d_{ij}”, “max_j(d_{ij})” and “min_j(d_{ij})”.

Response : We have modified, “xij, max(xj), min(xj)” are “dij”, “max(dj)” and “min(dj)”

Point: 5. Page 12, formula (14), what does the symbols “f_i” and “z_{ij}” stand for?

Response : fi is comprehensive weighted score, is the evaluation matrix after standardization.

Point: 6. Page 12, formula (15), “where \rho reflects the decision-maker’s preference for objective or subjective weight.” Does it reflect the preference of decision makers for objective or subjective weights here? The higher the value of \rho is, does the decision maker prefer subjective weight or objective weight?“where\rho

Response : where reflects the decision-maker’s preference for subjective weight. The higher the value of ρ is the decision maker prefer subjective weight.

Point: 7. Page 12, in theorem 1, “formula (10)” should be “formula (15)”.

Response : We have modified “formula (10)” for “formula (15)”. Also we have modified “From formula (12)” for “From formula (27)”.

Point: 8. Page 13, in step 3, “formula (18)” should be “formula (16)”.

Response : We have modified “formula (18)” for “formula (16)”.

Point: 9. It is suggested that the authors compare the proposed comprehensive weight method with existing comprehensive weight methods.

Response：Revised according to comments.

Point: 10. It is suggested that the authors add a sensitivity analysis with variable \rho.

Response：The sensitivity analysis with variable Rho is added according to the comments. After analysis, the variable Rho directly affects the closeness and project selection.
